# Efficacy and safety of ascending doses of praziquantel against *Schistosoma haematobium* infection in preschool-aged and school-aged children: a single-blind randomised controlled trial

**DOI:** 10.1186/s12916-018-1066-y

**Published:** 2018-06-01

**Authors:** Jean T. Coulibaly, Gordana Panic, Richard B. Yapi, Jana Kovač, Beatrice Barda, Yves K. N’Gbesso, Jan Hattendorf, Jennifer Keiser

**Affiliations:** 1 Department of Medical Parasitology and Infection Biology, Swiss Tropical and Public Health Institute, P.O. Box, CH-4002, Basel, Switzerland; 2 University of Basel, Basel, Switzerland; 3 Unité de Formation et de Recherche Biosciences, Université Félix Houphouët-Boigny, Abidjan, Côte d’Ivoire; 4 Centre Suisse de Recherches Scientifiques, Abidjan, Côte d’Ivoire; 5 Departement d’Agboville, Centre de Santé Urbain d’Azaguié, Azaguié, Côte d’Ivoire; 6 Department of Epidemiology and Public Health, Swiss Tropical and Public Health Institute, Basel, Switzerland

**Keywords:** Efficacy, Praziquantel, Preschool-aged children, S*chistosoma haematobium*, School-aged children

## Abstract

**Background:**

Despite decades of experience with praziquantel treatment in school-aged children (SAC) and adults, we still face considerable knowledge gaps relevant to the successful treatment of preschool-aged children (PSAC). This study aimed to assess the efficacy and safety of escalating praziquantel dosages in PSAC infected with *Schistosoma haematobium*.

**Methods:**

We conducted a randomised, dose-finding trial in PSAC (2–5 years) and as comparator a cohort of SAC (6–15 years) infected with *S. haematobium* in Côte d’Ivoire. A total of 186 PSAC and 195 SAC were randomly assigned to 20, 40 or 60 mg/kg praziquantel or placebo. The nature of the dose-response relationship in terms of cure rate (CR) was the primary objective. Egg reduction rate (ERR) and tolerability were secondary outcomes. CRs and ERRs were assessed using triplicate urine filtration over 3 consecutive days. Available-case analysis was performed including all participants with primary endpoint data.

**Results:**

A total of 170 PSAC and 174 SAC received treatment. Almost 90% of PSAC and three quarters of SAC were lightly infected with *S. haematobium*. Follow-up data were available for 157 PSAC and 166 SAC. In PSAC, CRs of praziquantel were 85.7% (30/35), 78.0% (32/41) and 68.3% (28/41) at 20, 40 and 60 mg/kg and 47.5% (19/40) for placebo. In SAC, CRs were 10.8% for placebo (4/37), 55.6% for 20 mg/kg (25/45), 68.3% for 40 mg/kg (28/41) and 60.5% for 60 mg/kg (26/43). ERRs based on geometric means ranged between 96.5% (60 mg/kg) and 98.3% (20 mg/kg) in PSAC and between 97.6% (20 mg/kg and 60 mg/kg) and 98.6% (40 mg/kg) in SAC. Adverse events were mild and transient.

**Conclusions:**

Praziquantel revealed dose-independent efficacy against light infections of *S. haematobium*. Over the dose range tested, praziquantel displayed a ceiling effect with the highest response for 20 mg/kg in PSAC. In SAC maximum efficacy was obtained with 40 mg/kg praziquantel. Further investigations are required in children with moderate to heavy infections.

**Trial registration:**

This trial is registered with International Standard Randomised Controlled Trial Number ISRCTN15280205.

**Electronic supplementary material:**

The online version of this article (10.1186/s12916-018-1066-y) contains supplementary material, which is available to authorized users.

## Background

Schistosomiasis is a major public health problem with an estimated 779 million people at risk of infection [1]. The disease is caused by trematode worms of the genus *Schistosoma*, where infection with *Schistosoma japonicum* and *S. mansoni* causes mostly intestinal schistosomiasis, while *S. haematobium* is responsible for genitourinary schistosomiasis [2–6]. Cumulative schistosome infections over years, due to rapid reinfection, result in morbid sequelae, including haematuria, nutritional deficiencies, anaemia, hepatic peri-portal fibrosis and consequent portal hypertension and delayed physical and cognitive development [7–9]. Moreover, genitourinary schistosomiasis can lead to obstruction and carcinomas of urogenital organs and impairment to female reproductive health [6, 10]. To control schistosomiasis morbidity, health authorities rely on mass administration (preventive chemotherapy) of praziquantel in school-aged children (SAC), the population most affected [11–13]. In 2010, the World Health Organization (WHO) endorsed the inclusion of preschool-aged children (PSAC) in preventative chemotherapy programmes, since there is increasing evidence that they are also affected by schistosomiasis and could suffer from morbidity [14–17]. In the absence of an appropriate paediatric formulation, broken or crushed praziquantel tablets are commonly used in PSAC using the standard 40 mg/kg dose [18]. A range of studies showed that this dose was well tolerated and efficacious [15, 19, 20]. However, the heterogeneity of methodology and reporting on praziquantel efficacy and safety assessment make decision-making difficult [21].

In the paediatric population, growth and maturation of organs are dynamic. Changes in body proportion and metabolism occur throughout infancy and childhood that affect how drugs are metabolised [22, 23]. Well-designed paediatric drug trials are therefore warranted in order to guide the proper usage of drug treatments to avoid underdosing, overdosing, ineffectiveness and safety problems.

We recently conducted a randomised, controlled dose-finding study assessing the safety and efficacy of praziquantel in PSAC and SAC infected with *S. mansoni.* Considerable differences were observed between these two age groups with regard to efficacy. For example, while treatment of SAC with 40 or 60 mg/kg met the WHO standards of clinical efficacy of ≥ 90% egg reduction rate (ERR) based on arithmetic mean (AM), none of the doses administered could reach this threshold in PSAC [24].

This study was designed to support the ongoing efforts to successfully control schistosome infections in PSAC by assessing the efficacy and safety of escalating praziquantel dosages in PSAC compared to SAC infected with *S. haematobium*. The clinical evidence for praziquantel obtained for both *S. haematobium* and *S. mansoni* in PSAC will facilitate the clinical decision-making process, resulting in successful control of schistosome infection and disease.

## Methods

### Study design and participants

We conducted a randomised, parallel-group, single-blind, placebo-controlled, dose-ranging trial between November 2015 and February 2016. PSAC (aged 2–5 years) and SAC (6–15 years) were surveyed in five villages of the health district of Adzopé, southern Côte d’Ivoire. In total, 740 PSAC and 444 SAC were registered during the census and were invited to participate in the study.

### Randomisation and masking

Eligibility of children was based on the presence of *S. haematobium* eggs in their urine. In addition, a clinical examination and an oral medical history by active questioning were implemented in order to exclude children with abnormal medical conditions (i.e. clinical malaria or hepato-splenic schistosomiasis) or those who received an antimalarial or anthelmintic drug in the past 4 weeks.

*S. haematobium* egg-positive PSAC and SAC, eligible for the study, were stratified according to baseline infection intensity into light (< 50 eggs/10 mL of urine) or heavy (≥ 50 eggs/10 mL of urine) infection intensities [25]. Children were then randomly assigned to placebo or 20, 40 or 60 mg/kg praziquantel treatment arms using computer-generated stratified block randomisation codes provided by an independent statistician based on the aforementioned infection intensity (block size of 8). Only the investigator dealing with drug administration was aware of the treatment assignments. The physician and laboratory technicians were blinded to the treatment. SAC might have recognised the treatment dose due to the number of tablets administered; however, the crushing of tablets for PSAC was prepared in advance. Masking was maintained throughout the trial. Randomisation codes were released after the database was unlocked.

### Field and laboratory procedures

During the baseline survey, three urine samples over 3 consecutive days and a single stool sample from the first collection day were collected between 10:00 and 14:00 am from each participating child. Urine and stool samples were transferred to a nearby laboratory in Azaguié town and examined on the day of collection. *S. haematobium* was detected using the urine filtration method (syringe filtration of 10 mL of urine followed by microscopic examination of the filter) [26]. A subsequent independent quality control of sample results (approximately 10%) was conducted. If a difference in presence/absence of *S. haematobium* eggs was observed or egg counts exceeded +/−10 eggs for light infections or +/−20 eggs for heavy infections, all the slides were read once again by the senior technician. *S. mansoni* infection was assessed through duplicate Kato-Katz thick smears (standard template of 41.7 mg) [27]. Eggs of soil-transmitted helminths, i.e. *Ascaris lumbricoides*, hookworm and *Trichuris trichiura*, were also assessed and recorded for each parasite species separately. Moreover, finger prick blood samples were taken to assess *Plasmodium* infections and haemoglobin amount using, respectively, thick and thin blood smears [24] and a calibrated HemoCue device (HemoCue 301 system, HemoCue, Ängelholm, Sweden).

To assess treatment efficacy, another three urine samples and a single stool sample were collected between 21 and 25 days post-treatment and subjected to the same diagnostic approaches applied at baseline. At the end of the study, all children enrolled in the study were offered albendazole (400 mg) and praziquantel (40 mg/kg) for the treatment of helminth infections according to local guidelines.

### Treatment

Prior to treatment, each child received breakfast. In both study groups (SAC and PSAC), treatment was done based on the child’s body weight (graduated increments of 0.1 kg). Praziquantel (600 mg Cesol®) (used in quarter-tablet increments) and placebo were obtained from Merck KGaA, Darmstadt, Germany and Fagron, Barsbüttel, Germany, respectively. For PSAC, tablets were crushed using a mortar and pestle and dissolved in a small volume of syrup-flavoured water to mask the taste. SAC and the mothers/guardians of PSAC were interviewed 3, 24, 48 and 72 h after treatment for adverse events and the intensities graded by the study physician as mild, moderate, severe or intolerable [24]*.*

### Outcomes and sample size determination

The cure rate (CR) (primary outcome) was expressed as the proportion of children positive for *S. haematobium* eggs at baseline survey who became negative at follow-up. The secondary outcomes were ERR and the safety of different doses of praziquantel.

Simulations showed that with 40 children enrolled per treatment arm (0, 20, 40 and 60 mg/kg), the dose-response prediction model should have a median precision—defined as one half length of the 95% confidence interval (CI)—of 10% points, assuming associated cure rates of 2.5%, 50%, 75% [28] and 90%.

### Statistical analysis

All data were first double entered into an Excel spreadsheet, then transferred into Epi Info version 3.5.2 (Centers for Disease Control and Prevention, Atlanta, GA, USA) and cross-checked. R version 3.4.0 was used for all statistical analyses. Available-case analysis was implemented including all treated participants (regardless of whether they could swallow the drug or not or were wrongly dosed) who had at least one urine sample examined with the urine filtration method at follow-up and were not excluded due to a medical condition.

In order to calculate ERR, the AM and geometric mean (GM) of eggs per 10 mL of urine before and after treatment were assessed. Geometric mean egg counts were calculated as follows: e^1/n ∑ log(x + 1)^ -1, and the corresponding ERR ([1 – geometric mean egg output after treatment/geometric mean egg output at baseline] × 100) was assessed. A bootstrap resampling method with 5000 replicates was used to estimate 95% CIs for ERRs.

E_max_ models using the DoseFinding package (version 0∙9–14) of the statistical software environment R (v3.3.0) were implemented to predict the dose-response curves in terms of CRs and ERRs. Logistic regression was used to predict CR by infection intensity at baseline.

## Results

### Study flow and baseline characteristics

**Fig. 1:**
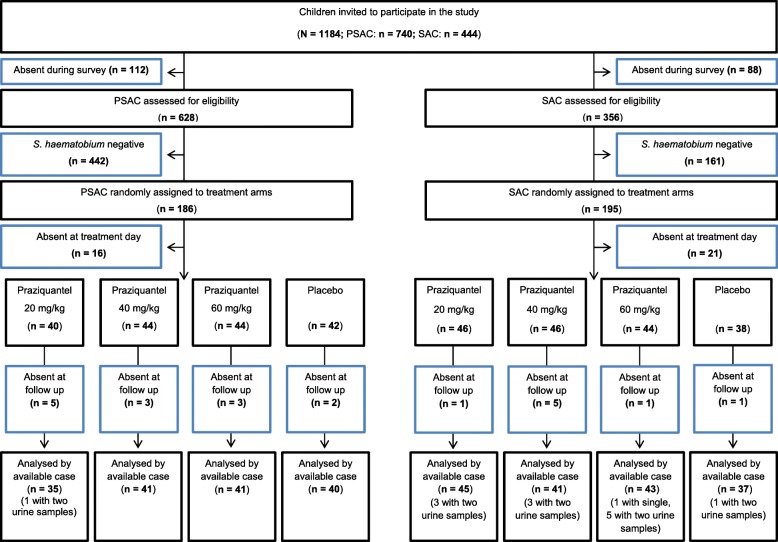
Trial profile

Overall, 1184 children were invited to participate in the study (Fig. 1). At baseline, 628 PSAC and 356 SAC were screened for *S. haematobium* infection. Of these, 186 (29.6%) PSAC and 195 (45.7%) SAC had a detectable *S. haematobium* infection and were randomised for treatment. On the treatment day, 16 PSAC and 21 SAC were absent. PSAC received 20 mg/kg (*n* = 40), 40 mg/kg (*n* = 44) or 60 mg/kg (*n* = 44) praziquantel or placebo (*n* = 42). SAC were likewise allocated to 20 mg/kg (*n* = 46), 40 mg/kg (*n* = 46) or 60 mg/kg praziquantel (*n* = 44) or placebo (*n* = 38). Two PSAC and one SAC were not able to swallow the drug. One PSAC and one SAC were wrongly dosed (62.5 mg/kg instead of 40 mg/kg and 75 mg/kg instead of 40 mg/kg, respectively).

At follow-up, data were available for 157 PSAC and 166 SAC. One PSAC and 12 SAC provided only two urine samples, while one SAC provided only one urine sample.

The median age, weight, height and sex of PSAC and SAC were balanced among the treatment groups (Table 1). Three quarters of PSAC and SAC were lightly infected with *S. haematobium*. No infection with *A. lumbricoides*, *T. trichiura* or hookworm was recorded. Co-infections among *S. haematobium*-infected children with *S. mansoni* and *P. falciparum* were very low (less than 9%) in PSAC and SAC. Median haemoglobin values ranged between 10.5 and 11.0 g/dL in PSAC and between 11.0 and 11.7 g/dL in SAC.

**Table 1:** Baseline characteristics

	Preschool-aged children (PSAC)				School-aged children (SAC)			
	Treatment arm				Treatment arm			
Characteristics	Placebo	20 mg/kg	40 mg/kg	60 mg/kg	Placebo	20 mg/kg	40 mg/kg	60 mg/kg
	42	40	44	44	38	46	46	44
Female *N* (%)	23 (54.8)	21 (52.5)	20 (45.5)	27 (61.4)	23 (60.5)	25 (54.3)	25 (54.3)	25 (56.8)
Age, years; median	4	4	4	4	9	8	8	9
[IQR]	[2–5]	[2–5]	[2–5]	[2–5]	[6–13]	[6–13]	[6–14]	[6–13]
Weight, kg; median	15	15	15	15	22	22	24	22
[IQR]	[10–21]	[11–19]	[11–19]	[11–18]	[18–35]	[18–33]	[18–38]	[18–40]
Height, cm; median	97	98	101	100	125	125	125	124
[IQR]	[80–117]	[83–115]	[84–116]	[83–114]	[109–141]	[114–139]	[113–149]	[112–150]
Haemoglobin (g/dL); median	10.5	11.0	10.9	10.9	11.4	11.2	11.7	11.6
[IQR]	[9.1–13.2]	[9.1–12.8]	[8.8–12.5]	[8.5–12.9]	[9.7–13.7]	[9.9–12.4]	[9.7–12.8]	[10.1–13.5]
Infection intensity *N* (%)								
Light	36 (85.7)	38 (95.0)	38 (86.4)	40 (90.9)	27 (71.1)	35 (76.1)	36 (78.3)	33 (75.0)
Heavy	6 (14.3)	2 (5.0)	6 (13.6)	4 (9.1)	11 (28.9)	11 (23.9)	10 (21.7)	11 (25.0)
Co-infections *N* (%)								
*S. mansoni*	0 (0.0)	0 (0.0)	1 (2.3)	1 (2.3)	0 (0.0)	1 (2.2)	1 (2.2)	0 (0.0)
*Plasmodium falciparum*	1 (2.4)	0 (0.0)	1 (2.3)	0 (0.0)	1 (2.6)	4 (8.7)	2 (4.3)	3 (6.8)
(based on thin/thick smear)								

*IQR* interquartile range

### Efficacy of praziquantel

**Fig. 2:**
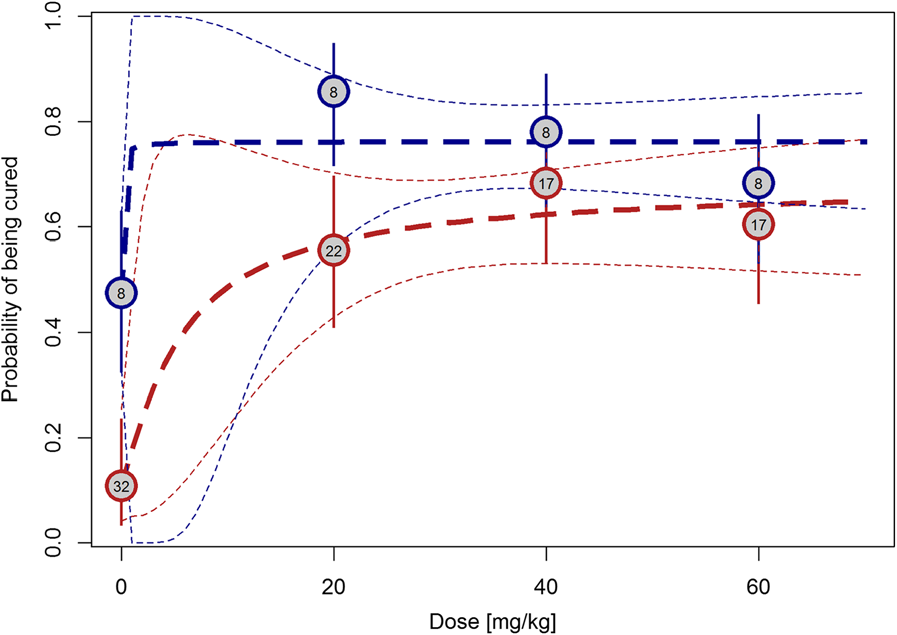
Cure rates in PSAC (*blue lines*) and SAC (*red lines*). *Circles* show observed cure rates with 95% CIs (*vertical lines*). *Numbers in the circles* show geometric mean infection intensities at baseline (BL). *Dashed lines* represent the estimated dose-response curve and corresponding 95% CIs predicted by the E_max_ models. Geometric mean of infection intensity was the mean of eggs filtered from 10 mL of urine

The nature of the dose response based on CRs is depicted in Fig. 2. Praziquantel revealed dose-independent efficacy with the highest cure rates observed at 20 and 40 mg/kg in PSAC and SAC, respectively. The E_max_ model based on actual doses on the per protocol population is presented in Additional file 1: Figure S1 and shows a similar trend. Additional file 1: Figure S2 presents the predicted probability of being cured by baseline infection intensity. For all treatments, including placebo, there was a high probability of being cured at low infection intensities.

CRs in PSAC for 20 mg/kg, 40 mg/kg and 60 mg/kg were 85.7 (95% CI 69.7–95.2), 78.0% (95% CI 62.4–89.4) and 68.3% (95% CI 51.9–81.9), respectively, whereas in SAC the respective CRs were 55.6% (95% CI 40.0–70.4), 68.3% (95% CI 51.9–81.9) and 60.5% (95% CI 44.4–75.0). In the placebo groups, *S. haematobium* eggs were not detected in the urine samples of 47.5% (19/40) and 10.8% (4/37) in PSAC and SAC, respectively (Table 2). Imputation of missing data with treatment failure or success in the intention-to-treat analysis did not change the observed outcomes (Additional file 1: Table S1).

**Table 2:** Available-case analysis of cure and egg reduction rates of 20, 40 and 60 mg/kg praziquantel versus placebo against urogenital schistosomiasis in PSAC and SAC based on the urine filtration method

	Preschool-aged children (PSAC)				School-aged children (SAC)			
	Placebo	20 mg/kg	40 mg/kg	60 mg/kg	Placebo	20 mg/kg	40 mg/kg	60 mg/kg
Infected children before treatment (*N*)	40	35	41	41	37	45	41	43
Actual dose administered (range; mg/kg)	–	13.6–25	34.6–62.5^a^	50–70	–	16.7–23.7	36.4–75^b^	56.3–65.6
Cured children after treatment *N* (%)	19 (47.5)	30 (85.7)	32 (78.0)	28 (68.3)	4 (10.8)	25 (55.6)	28 (68.3)	26 (60.5)
95% CI	32.5–63.9	69.7–95.2	62.4–89.4	51.9–81.9	3.0–25.4	40.0–70.4	51.9–81.9	44.4–75.0
Cured children according to sex								
Male	6 (31.6)	14 (46.7)	19 (59.4)	10 (35.7)	1 (25.0)	9 (36.0)	13 (46.4)	9 (34.6)
95% CI	12.5–56.6	28.3–65.7	40.6–76.3	18.6–55.9	0.6–80.6	18.0–57.5	27.5–66.1	17.2–55.7
Female	13 (68.4)	16 (53.3)	13 (40.6)	18 (64.3)	3 (75.0)	16 (64.0)	15 (53.6)	17 (65.4)
95% CI	43.4–87.4	34.3–71.7	23.7–59.4	44.1–81.4	19.4–99.4	42.5–82.0	33.9–72.5	44.3–82.8
Cured children with light infection	19/40 (47.5)	29/33 (87.9)	28/35 (80.0)	27/37 (73.0)	4/26 (15.4)	24/34 (70.6)	26/33 (78.8)	23/32 (71.9)
Cured children with heavy infections (%)	0/6 (0)	1/2 (50.0)	4/6 (66.7)	1/4 (25.0)	0/11 (0.0)	1/11 (9.1)	2/8 (25.0)	3/11 (27.3)
Geometric mean eggs/10 mL of urine								
Before treatment	7.8	7.5	8.4	8.3	31.5	21.6	16.6	17.2
After treatment	2.4	0.1	0.2	0.3	13.1	0.5	0.2	0.4
Egg reduction rate	68.9	98.3	97.6	96.5	58.5	97.6	98.6	97.6
(95% CI)	46.6–83.6	95.4–99.8	94.9–99.2	93.1–98.7	38.7–71.4	96.4–98.6	97.7–99.3	95.3–98.9
Arithmetic mean eggs/10 mL of urine								
Before treatment	22.7	14.3	21.7	16.9	94.4	89.5	31.0	34.4
After treatment	11.5	0.3	0.4	0.9	49.0	1.2	0.4	1.0
Egg reduction rate	49.5	97.8	98.2	94.5	46.9	98.7	98.8	97.0
(95% CI)	0.2–77.3	93.6–99.9	96.1–99.5	85.7–99.1	36.4–77.6	96.7–99.3	97.7–99.5	92.9–99.2

^a^Range 34.6–44.1 excluding the wrongly dosed child

^b^Range 36.4–42.9 excluding the wrongly dosed child

**Fig. 3:**
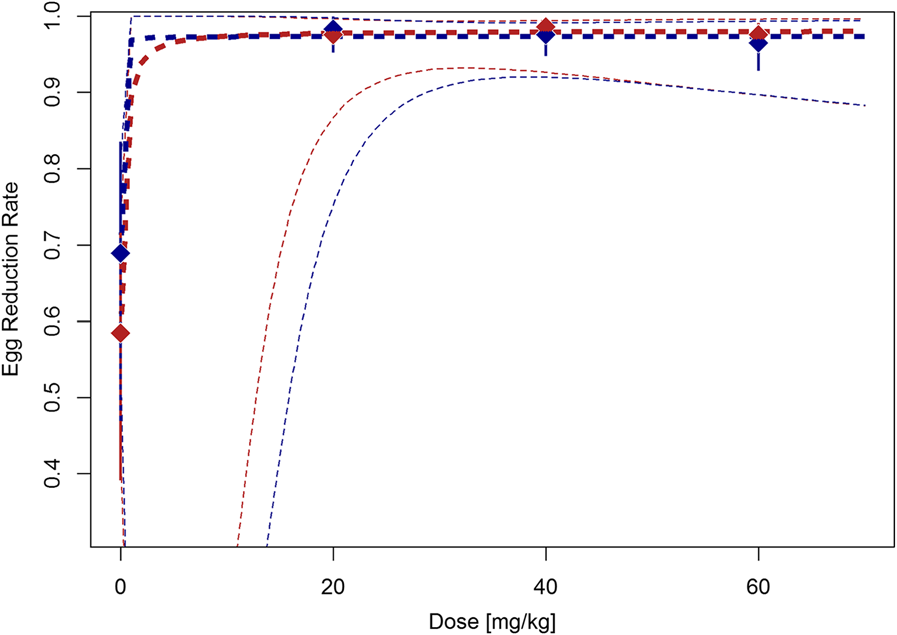
Egg reduction rates in PSAC (*blue lines*) and SAC (*red lines*). *Diamonds* show observed cure rates with 95% CIs (*vertical lines*). *Dashed lines* represent the estimated dose-response curve and corresponding 95% CI predicted by the E_max_ model

ERRs are summarized in Table 2 and depicted in Fig. 3. ERRs in PSAC were 98.3% for 20 mg/kg, 97.6% for 40 mg/kg and 96.5% for 60 mg/kg. In SAC ERRs of 97.6%, 98.6% and 97.6% were observed with increasing dosages. ERRs based on AMs had similar profiles to those based on GMs and are presented in Table 2. Table 2 also presents an exploratory subgroup analysis on CRs according to *S. haematobium* infection intensity. The CR in PSAC ranged from 73.0% (60 mg/kg) to 87.9% (20 mg/kg) in light infections and from 25.0% (60 mg/kg) to 66.7% (40 mg/kg) in heavy infections. In SAC, CRs were 70.6% (20 mg/kg) to 78.8% (40 mg/kg) for light *S. haematobium* infections and between 9.1% (20 mg/kg) and 27.3% (60 mg/kg) for heavy infections.

### Safety of praziquantel

Adverse events data were available for 168 PSAC and 173 SAC (Table 3). In both groups, more children reported signs and symptoms at pre-treatment compared to 3 and 24 h post-treatment. No serious adverse events were reported. Overall, adverse events were mild with fewer adverse events observed at 3 h post-treatment compared to pre-treatment in PSAC (52 episodes versus 88 episodes) and in SAC (88 episodes versus 92 episodes), respectively. Mild events mainly included fever, headache, nausea, diarrhoea, vomiting, dizziness and stomach ache. Few moderate cases were reported at 3 h after treatment in PSAC (only one with moderate diarrhoea) and in SAC (*n* = 12). At 24 h post-treatment, 25 (14.9%) and 47 (27.2%) adverse events were recorded in PSAC and SAC, respectively. For both age groups the number of adverse events was similar among the three praziquantel treatment arms, with fewer adverse events observed in the placebo-treated groups. The most common adverse events in PSAC and SAC 24 h post-treatment were diarrhoea (4.8 and 3.5%), stomach ache (3.6 and 9.8%), fever (6.0 and 13.3%), headache (3.0 and 15.6%) and nausea (2.4 and 6.9%).

**Table 3:** Main type of clinical symptoms (number and percentage) before treatment and adverse events 3 and 24 h after praziquantel administration in *Schistosoma haematobium*-infected preschool-aged children (*n* = 168) and school-aged children (*n* = 173)

	Preschool-aged children (PSAC)^a^					School-aged children (SAC)^b^				
Symptoms	Placebo	20 mg/kg	40 mg/kg	60 mg/kg	Overall	Placebo	20 mg/kg	40 mg/kg	60 mg/kg	Overall
	(*n* = 41)	(n = 40)	(n = 44)	(*n* = 43)	(*n* = 168)	(*n* = 38)	(*n* = 45)	(*n* = 46)	(*n* = 44)	(*n* = 173)
Before treatment										
Moderate	0 (0.0)	0 (0.0)	0 (0.0)	0 (0.0)	0 (0.0)	0 (0.0)	0 (0.0)	0 (0.0)	0 (0.0)	0 (0.0)
Mild	24 (58.5)	19 (47.5)	26 (59.1)	19 (44.2)	88 (52.4)	20 (52.6)	26 (57.8)	25 (54.3)	21 (47.7)	92 (53.2)
None	17 (41.5)	21 (52.5)	18 (40.9)	24 (55.8)	80 (47.6)	18 (47.4)	19 (42.2)	21 (45.7)	23 (52.3)	81 (46.8)
Fever	6 (14.1)	3 (7.5)	7 (15.9)	5 (11.6)	21 (12.5)	5 (13.2)	7 (15.6)	5 (10.9)	6 (13.6)	23 (13.3)
Headache	8 (19.5)	6 (15.0)	13 (29.5)	10 (23.3)	37 (22.0)	3 (7.9)	7 (15.6)	10 (21.7)	7 (15.9)	27 (15.6)
Nausea	3 (7.3)	4 (10.0)	3 (6.8)	1 (2.3)	11 (6.5)	2 (5.3)	1 (2.2)	4 (8.7)	5 (11.4)	12 (6.9)
Vomiting	1 (2.4)	1 (2.5)	1 (2.3)	2 (4.7)	5 (3.0)	0 (0.0)	0 (0.0)	1 (2.2)	0 (0.0)	1 (0.6)
Diarrhoea	4 (9.6)	4 (10.0)	4 (9.1)	5 (11.6)	17 (10.1)	0 (0.0)	1 (2.2)	3 (6.5)	2 (4.5)	6 (3.5)
Dizziness	2 (4.9)	0 (0.0)	0 (0.0)	0 (0.0)	2 (1.2)	0 (0.0)	0 (0.0)	0 (0.0)	0 (0.0)	0 (0.0)
Stomach ache	2 (4.9)	5 (12.5)	8 (18.2)	4 (9.3)	19 (11.3)	1 (2.6)	6 (13.3)	6 (13.0)	4 (9.1)	17 (9.8)
3 h post-treatment										
Moderate	0 (0.0)	0 (0.0)	0 (0.0)	1 (2.3)	1 (0.6)	1 (2.6)	2 (4.4)	4 (8.7)	5 (11.4)	12 (6.9)
Mild	6 (14.6)	8 (20.0)	15 (34.1)	23 (53.5)	52 (31.0)	18 (47.4)	23 (51.1)	21 (45.7)	26 (59.1)	88 (50.9)
None	35 (85.4)	32 (80.0)	29 (65.9)	19 (44.2)	115 (68.5)	19 (50.0)	20 (44.4)	21 (45.7)	13 (29.5)	73 (42.2)
Fever	3 (7.3)	3 (7.5)	2 (4.5)	4 (9.3)	12 (7.1)	6 (15.8)	4 (8.9)	8 (17.4)	12 (27.3)	30 (17.3)
Headache	1 (2.4)	2 (5.0)	1 (2.3)	7 (16.3)	11 (6.5)	8 (21.1)	10 (22.2)	6 (13.0)	6 (13.6)	30 (17.3)
Nausea	1 (2.4)	1 (2.5)	4 (9.1)	5 (11.6)	11 (6.5)	4 (10.5)	3 (6.7)	9 (19.6)	14 (31.8)	30 (17.3)
Vomiting	0 (0.0)	1 (2.5)	3 (6.8)	9 (20.9)	13 (7.7)	1 (2.6)	3 (6.7)	8 (17.4)	10 (22.7)	22 (12.7)
Diarrhoea	0 (0.0)	1 (2.5)	1 (2.3)	3 (7.0)	5 (3.0)	3 (7.9)	3 (6.7)	2 (4.3)	0 (0.0)	8 (4.6)
Dizziness	1 (2.4)	1 (2.5)	2 (4.5)	4 (9.3)	8 (4.8)	5 (13.2)	4 (8.9)	8 (17.4)	5 (11.4)	22 (12.7)
Stomach ache	3 (7.3)	4 (10.0)	4 (9.1)	2 (4.7)	13 (7.7)	5 (13.2)	11 (24.4)	9 (19.6)	12 (27.3)	37 (21.4)
24 h post-treatment										
Moderate	0 (0.0)	0 (0.0)	0 (0.0)	0 (0.0)	0 (0.0)	0 (0.0)	0 (0.0)	0 (0.0)	0 (0.0)	0 (0.0)
Mild	4 (9.6)	7 (17.5)	8 (18.2)	6 (14.0)	25 (14.9)	12 (31.6)	12 (26.7)	12 (26.1)	11 (25.0)	47 (27.2)
None	37 (90.2)	33 (82.5)	36 (81.8)	37 (86.0)	143 (85.1)	26 (68.4)	33 (73.3)	34 (73.9)	33 (75.0)	126 (72.8)
Fever	2 (4.9)	4 (10.0)	2 (4.5)	2 (4.7)	10 (6.0)	5 (13.2)	7 (15.6)	5 (10.9)	6 (13.6)	23 (13.3)
Headache	1 (2.4)	1 (2.5)	1 (2.3)	2 (4.7)	5 (3.0)	3 (7.9)	7 (15.6)	10 (21.7)	7 (15.9)	27 (15.6)
Nausea	1 (2.4)	0 (0.0)	0 (0.0)	3 (7.0)	4 (2.4)	2 (5.3)	1 (2.2)	4 (8.7)	5 (11.4)	12 (6.9)
Vomiting	0 (0.0)	0 (0.0)	0 (0.0)	3 (7.0)	3 (1.8)	0 (0.0)	0 (0.0)	1 (2.2)	0 (0.0)	1 (0.6)
Diarrhoea	1 (2.4)	2 (5.0)	5 (11.4)	0 (0.0)	8 (4.8)	0 (0.0)	1 (2.2)	3 (6.5)	2 (4.5)	6 (3.5)
Dizziness	1 (2.4)	0 (0.0)	0 (0.0)	1 (2.3)	1 (0.6)	0 (0.0)	0 (0.0)	0 (0.0)	0 (0.0)	0 (0.0)
Stomach ache	2 (4.9)	0 (0.0)	2 (4.5)	2 (4.7)	6 (3.6)	1 (2.6)	6 (13.3)	6 (13.0)	4 (9.1)	17 (9.8)

^a^2 children were absent (placebo (*n* = 1) and 60 mg/kg (*n* = 1)) following treatment and were not assessed for adverse events

^b^1 child was absent (20 mg/kg treatment arm) following treatment and was not assessed for adverse events

## Discussion

Over the past decade, preventive chemotherapy programmes for the control of schistosomiasis targeting SAC have scaled up across many countries in tropical and subtropical areas. Great progress has been made in decreasing the burden of this disease [29–31]. However, recent modelling and health economic studies found that expanded community-wide preventive chemotherapy that includes adolescents, adults and PSAC would better reduce the overall disease burden, rates of transmission and reinfection [32].

It was recommended in 2010 that PSAC should be included in preventive chemotherapy programmes [33] using an adequate dose, though this age group is still lacking a suitable formulation. A paediatric formulation of praziquantel (small, orally dispersible tablets) is under development (https://www.pediatricpraziquantelconsortium.org/node/28), but it will take several more years until the drug is marketed and available to all PSAC. To be able to treat preschoolers safely and effectively, we studied ascending doses of praziquantel in PSAC and SAC infected with *S. haematobium.* Our results build on an earlier dose-finding study in *S. mansoni*-infected children [24].

Several findings of our study are worth highlighting. First, the highest overall CRs among PSAC (85.7%) and SAC (68.3%) were obtained with 20 mg/kg and 40 mg/kg praziquantel, respectively and not with the highest dose administered, 60 mg/kg. For both age groups, CRs revealed even a slight inverse dose-rate effect. Similarly, ERRs increased very fast up to 98% and did not increase further regardless of the praziquantel dose administered. Interestingly, 60 mg/kg praziquantel also showed lower CRs in PSAC with moderate/high *S. haematobium* infection intensities compared to the two lower doses. For example, the CR in PSAC characterised by heavy infection intensities treated with 60 mg/kg praziquantel was as low as 25%. However, only a handful of PSAC suffered from moderate and high infection intensities; hence, no clear picture can be drawn for this age group. In SAC similar CRs were observed in children harbouring heavy infection intensities treated with 40 and 60 mg/kg. In summary, a high dose of praziquantel seems to have no additional benefit in the treatment of *S. haematobium* infections. This result is in contrast with our recent study, where we reported that in SAC infected with *S. mansoni*, CRs increased with higher doses of praziquantel [24], while only moderate CRs were observed in PSAC at all doses administered.

Overall higher CRs were observed in PSAC (68–86%) when compared to SAC (56–68%), which mirrors a recent meta-analysis by Zwang et al., where 40 mg/kg praziquantel cured 87.3% of *S. haematobium-*infected PSAC compared to 71.4% of SAC [34]. Nonetheless, our finding can most likely be explained with the lower infection intensities present in PSAC, as CRs in children characterised by heavy infection intensities were low. Hence, our results confirm the relationship between CRs and infection intensity observed in previous studies [35, 36]. Overall, the results emphasise the need for rigorous treatment programmes in settings with heavy infection of *S. haematobium*, since reductions in egg output significantly correlated with decreased morbidity [37, 38].

No dose-response relationship was observed for ERRs in both age groups above 20 mg/kg. This finding is in line with an earlier meta-analysis by Zwang et al. [34] which found no significant relationship for dose and ERR for any of the *Schistosoma* species. However, our dose-finding study in *S. mansoni*-infected children showed that higher ERRs (based on GMs) were observed in children treated with 40 and 60 mg/kg compared to 20 mg/kg [24]. Recent WHO Standard Operating Procedures have set a threshold of a 90% ERR based on AM for clinical efficacy and recommend that control programmes should investigate drug performance in populations where the ERR is lower [39]. Regardless of age group and whether GM or AM was used to determine ERRs, we found that all praziquantel doses used against *S. haematobium,* in contrast to preschoolers infected with *S. mansoni* [24], yielded ERRs above 90%. Despite the excellent efficacy of 20 mg/kg of praziquantel against light *S. haematobium* infections in this study, the use of two different doses, namely 20 mg/kg for *S. haematobium* and 40 mg/kg for *S. mansoni* in settings where *Schistosoma* species are overlapping, would raise logistical and operational challenges since control programmes are acting at large-scale levels such as district or country levels. Therefore, rigorous cost-effectiveness studies need to be implemented before a change of treatment guidelines could be considered. However, at a point-of-care level, using a test-and-treat approach, 20 mg/kg and 40 mg/kg could be recommended to treat PSAC for *S. haematobium* and *S. mansoni* infections, respectively.

In PSAC we observed a high CR in the placebo arm similar to what was observed in our *S. mansoni* study [24]. The probability of being cured for placebo-treated children was particularly high in children with low egg loads despite using a relatively strong diagnostic approach at baseline and follow-up by collecting per child three consecutive urine samples for each time point (baseline and follow-up). On the other hand, no cured individual was observed in placebo-treated children with heavy infection intensity at baseline. The high CR observed in the placebo treatment arm among PSAC was thus likely reflective of the low sensitivity of the urine filtration method for light infections [40, 41]. Our findings underscore the value of adding a placebo group in *Schistosoma* drug efficacy trials—the overestimation of CRs due to potential false negatives in light infections is visible. More importantly, our observations emphasise the need for *Schistosoma* species-related standard operating procedures including reliable diagnostic tools, suitable for drug efficacy assessment for low infection intensities [40–42].

With regard to safety outcomes, the main adverse events observed in both PSAC and SAC are in line with the adverse events reported in previous studies [16, 24, 34]. We observed an increase of adverse events severity that was proportional to praziquantel dose in SAC, while only one child showed moderate diarrhoea at the 60 mg/kg treatment dose in PSAC. However, as mentioned earlier, the accuracy of the adverse event severity assessment in PSAC is questionable, in particular for the less visible mild adverse events, since the reporting is done by the children’s mothers.

## Conclusions

Praziquantel showed a high response rate in PSAC and SAC infected with *S. haematobium*, with high efficacy observed already at 20 mg/kg, particularly in light infections. No benefit was observed using higher praziquantel doses in the current study. However, to be able to provide ultimate dosing recommendations of praziquantel for PSAC, additional studies might be required to support our conclusions, including pharmacokinetic studies and studies in PSAC suffering from moderate and heavy *S. haematobium* infections.

## Additional file

Additional file 1: **Table S1.** Imputation on cure rates of individuals lost after treatment in 20, 40 and 60 mg/kg praziquantel and placebo treatment arms among *Schistosoma haematobium*-infected preschool-aged children and school-aged children (intention-to-treat). **Figure S1.** E_max_ model predicting cure rates (CRs) based on actual doses in preschool-aged children (*blue symbols*) and school-aged children (*red symbols*). **Figure S2.** Predicted probability of being cured by baseline infection intensity in preschool children (*blue lines*) and school-aged children (*red lines*). (DOCX 231 kb)

## Abbreviations

AM: Arithmetic mean
CR: Cure rate
ERR: Egg reduction rate
GM: Geometric mean
PSAC: Preschool-aged children
SAC: School-aged children
WHO: World Health Organization
