# Supplementary material for: Efficacy and safety of ascending doses of praziquantel against Schistosoma haematobium infection in preschool-aged and school-aged children: a single-blind randomised controlled trial
Source: BMC Med. 2018 Jun 1;16:81. doi: 10.1186/s12916-018-1066-y (PMC5984412; doi:10.1186/s12916-018-1066-y)
Supplement: Supplementary file 1 — Table S1. Imputation on cure rates of individuals lost after treatment in 20, 40 and 60 mg/kg praziquantel and placebo treatment arms among Schistosoma haematobium-infected preschool-aged children and school-aged children (intention-to-treat). Figure S1. Emax model predicting cure rates (CRs) based on actual doses in preschool-aged children (blue symbols) and school-aged children (red symbols). Figure S2. Predicted probability of being cured by baseline infection intensity in preschool children (blue lines) and school-aged children (red lines). (DOCX 231 kb) [file 12916_2018_1066_MOESM1_ESM.docx]

**Efficacy and safety of ascending doses of praziquantel against *Schistosoma haematobium* infection in preschool-aged and school-aged children*:* a single-blind randomized controlled trial**

Jean T. Coulibaly^1,2,3^ , Gordana Panic^1,2^, Richard B. Yapi^,4^, Jana Kovač^1,2^, Beatrice Barda^1,2^, Yves K. N’Gbesso^5^, Jan Hattendorf^2,6^, Jennifer Keiser^1,2*^

1. Department of Medical Parasitology and Infection Biology, Swiss Tropical and Public Health Institute, Basel, Switzerland

2. University of Basel, Basel, Switzerland

3. Unité de Formation et de Recherche Biosciences, Université Félix Houphouët-Boigny, Abidjan, Côte d’Ivoire

4. Centre Suisse de Recherches Scientifiques, Abidjan, Côte d’Ivoire

5. Centre de Santé Urbain d’Azaguié, Departement d’Agboville, Azaguié, Côte d’Ivoire

6. Department of Epidemiology and Public Health, Swiss Tropical and Public Health Institute, Basel, Switzerland

**Table of contents**

Table S1: Missing value imputation (intention-to-treat analysis) Page 3

Figure S1 Emax model predicting cure rates Page 4

Figure S2 Predicted probability of being cured by baseline infection intensity Page 5

**Table S1**: Imputation on cure rates of individuals lost after treatment in 20, 40 and 60 mg/kg praziquantel and placebo treatment arms among *Schistosoma haematobium* infected preschool-aged children and school-aged children (intention-to-treat)

|  | **Preschool-aged children (PSAC)** | | | | **School-aged children (SAC)** | | | |
| --- | --- | --- | --- | --- | --- | --- | --- | --- |
|  | Placebo | 20 mg/kg | 40 mg/kg | 60 mg/kg | Placebo | 20 mg/kg | 40 mg/kg | 60 mg/kg |
| All children lost at follow up cured (%) | 50 | 87.5 | 79.5 | 69.8 | 13.2 | 56.5 | 71.7 | 61.4 |
| None of the children lost at follow up cured (%) | 45.2 | 75 | 72.7 | 65.1 | 10.5 | 54.3 | 60.9 | 59.1 |

**Figure S1** **Emax model predicting cure rates (CRs) based on actual doses in preschool-aged children (blue symbols) and school-aged children (red symbols).**

Cure rates in PSAC (blue lines) and SAC (red lines). Circles show observed cure rates. Numbers in the circles show geometric mean infection intensities at baseline (BL). Dashed lines represent the estimated dose–response curve and corresponding 95% CIs predicted by the Emax models. The diameter of the symbols indicates the number of participants in each category. Children are regrouped according to their actual dosage received rounded to one decimal. 2 children which were not able to swallow the tablets are considered as not treated in this analysis.


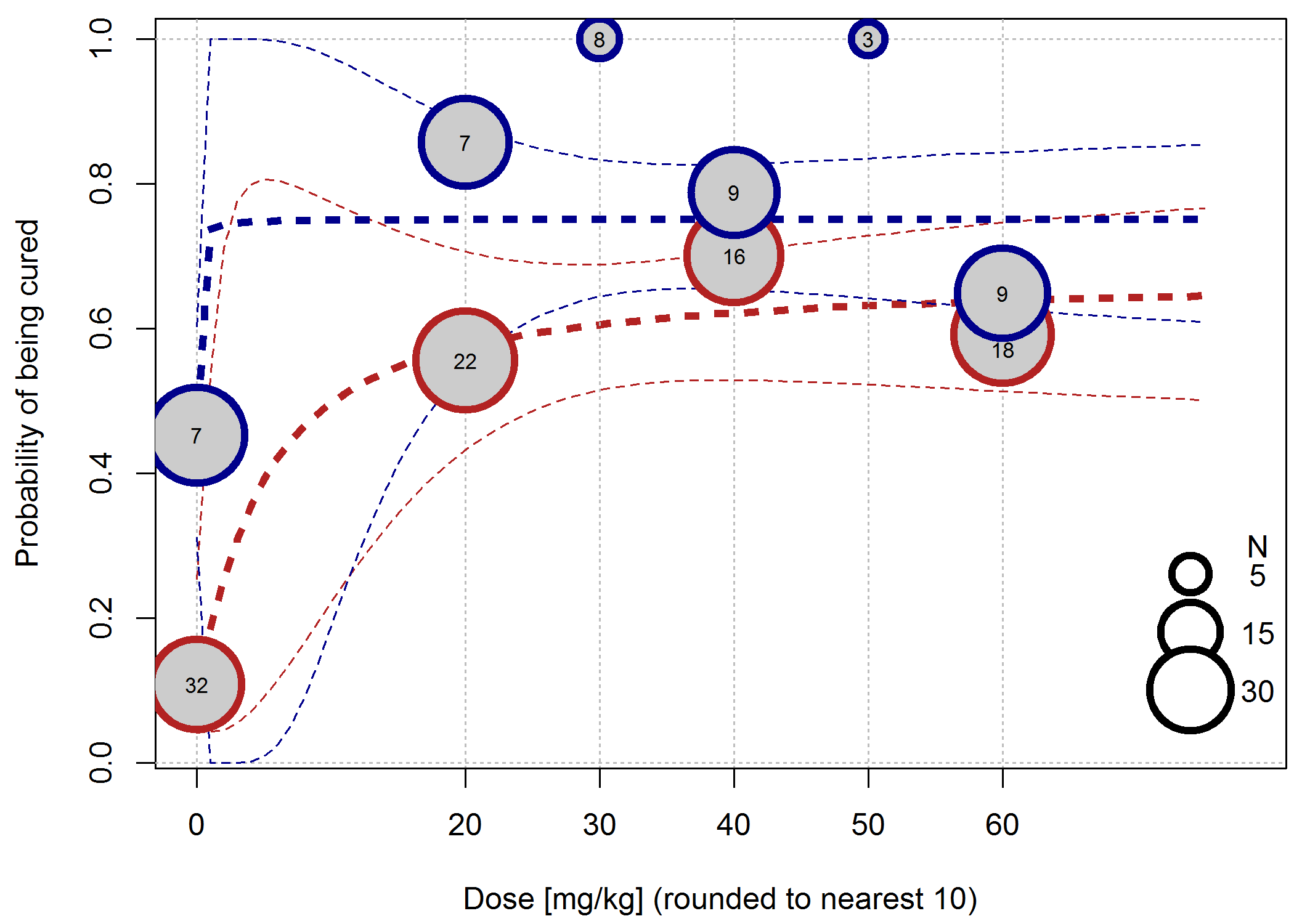


**Figure S2** **Predicted probability of being cured by baseline infection intensity in pre-school children (blue lines) and school age children (red lines).** Lines represent the predicted probabilities for the 8 treatment arms estimated by logistic regression. Circles represent predicted values of participants cured after treatment, stars indicate participants that remained infected after treatment. Darker symbols indicate higher infection intensity at follow up. Small random noise has been added to avoid overplotting.

**
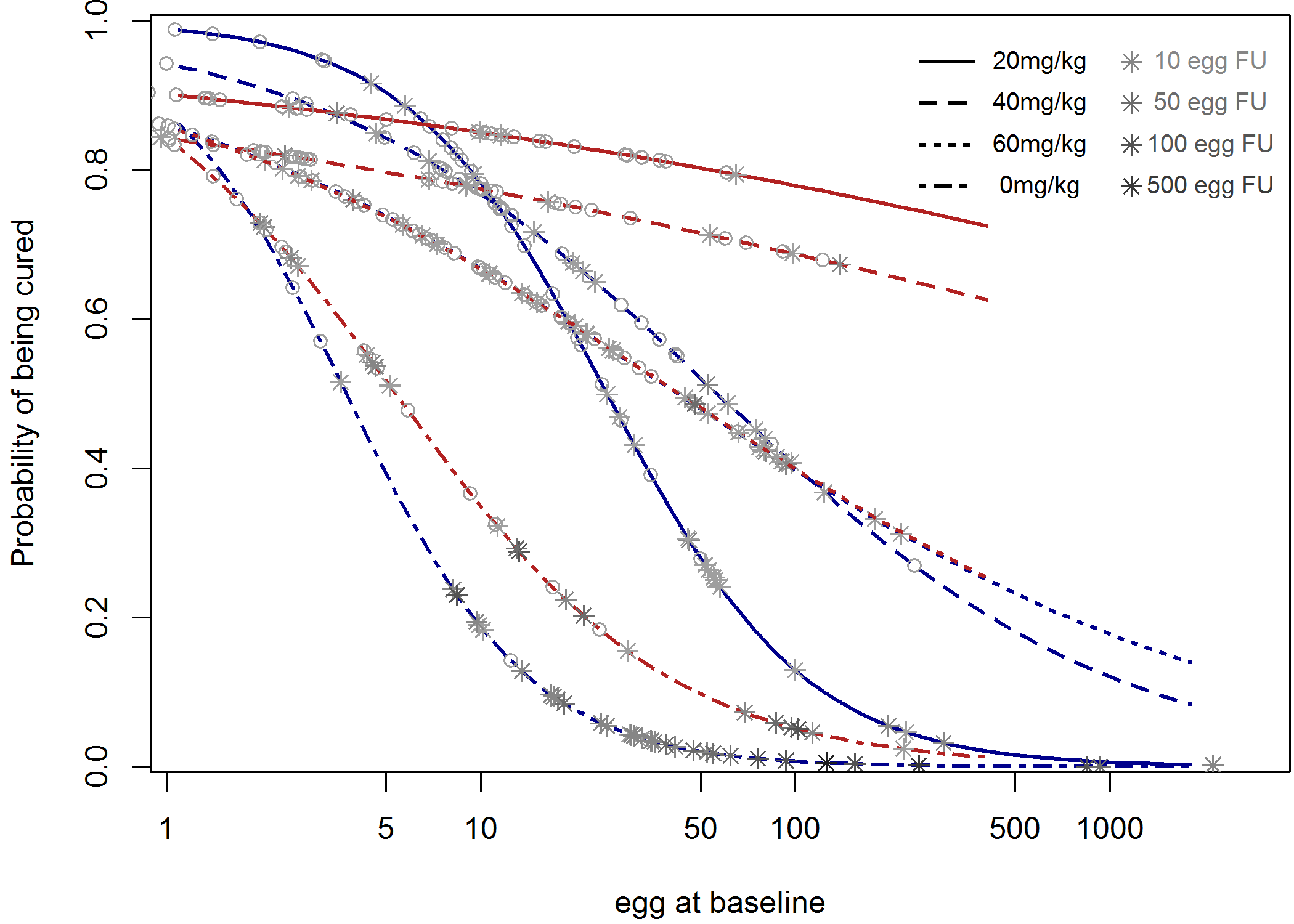
**
